# The Elicitin-Like Glycoprotein, ELI025, Is Secreted by the Pathogenic Oomycete *Pythium insidiosum* and Evades Host Antibody Responses

**DOI:** 10.1371/journal.pone.0118547

**Published:** 2015-03-20

**Authors:** Tassanee Lerksuthirat, Tassanee Lohnoo, Ruchuros Inkomlue, Thidarat Rujirawat, Wanta Yingyong, Rommanee Khositnithikul, Narumon Phaonakrop, Sittiruk Roytrakul, Thomas D. Sullivan, Theerapong Krajaejun

**Affiliations:** 1 Department of Pathology, Faculty of Medicine, Ramathibodi Hospital, Mahidol University, Bangkok, Thailand; 2 Research Center, Faculty of Medicine, Ramathibodi Hospital, Mahidol University, Bangkok, Thailand; 3 Molecular Medicine Program, Multidisciplinary Unit, Faculty of Science, Mahidol University, Bangkok, Thailand; 4 Proteomics Research Laboratory, Genome Institute, National Science and Technology Development Agency, Pathum Thani, Thailand; 5 Department of Pediatrics, School of Medicine and Public Health, University of Wisconsin, Madison, Wisconsin, United States of America; Agriculture and Agri-Food Canada, CANADA

## Abstract

*Pythium insidiosum* is a unique oomycete that can infect humans and animals. Patients with a *P*. *insidiosum* infection (pythiosis) have high rates of morbidity and mortality. The pathogen resists conventional antifungal drugs. Information on the biology and pathogenesis of *P*. *insidiosum* is limited. Many pathogens secrete proteins, known as effectors, which can affect the host response and promote the infection process. Elicitins are secretory proteins and are found only in the oomycetes, primarily in *Phytophthora* and *Pythium* species. In plant-pathogenic oomycetes, elicitins function as pathogen-associated molecular pattern molecules, sterol carriers, and plant defense stimulators. Recently, we reported a number of elicitin-encoding genes from the *P*. *insidiosum* transcriptome. The function of elicitins during human infections is unknown. One of the *P*. *insidiosum* elicitin-encoding genes, *ELI025*, is highly expressed and up-regulated at body temperature. This study aims to characterize the biochemical, immunological, and genetic properties of the elicitin protein, ELI025. A 12.4-kDa recombinant ELI025 protein (rELI025) was expressed in *Escherichia coli*. Rabbit anti-rELI025 antibodies reacted strongly with the native ELI025 in *P*. *insidiosum*’s culture medium. The detected ELI025 had two isoforms: glycosylated and non-glycosylated. ELI025 was not immunoreactive with sera from pythiosis patients. The region near the transcriptional start site of *ELI025* contained conserved oomycete core promoter elements. In conclusion, ELI025 is a small, abundant, secreted glycoprotein that evades host antibody responses. ELI025 is a promising candidate for development of diagnostic and therapeutic targets for pythiosis.

## Introduction

*Pythium insidiosum* is an organism that belongs to oomycetes, a group of fungus-like microorganisms [1]. While most of pathogenic oomycetes infect plants, *P*. *insidiosum* can infect humans and animals and cause a life threatening infectious disease, called pythiosis [1–4]. Although pythiosis is relatively rare compared to other infectious diseases, it has been increasingly reported from tropical and subtropical countries, such as, Brazil, Costa Rica, USA, Egypt, Mali, India, Malaysia, Thailand, Australia, and New Zealand [1–13]. Patients with pythiosis most commonly present with claudication and gangrenous ulcers of the lower extremities, as a result of chronic arterial infection and occlusive blood clots (vascular pythiosis) [4]. An alternative form, ocular pythiosis, presents with corneal ulcer and keratitis, as a result of ocular infection [4]. Pythiosis has a high rate of morbidity and mortality. Health care personnel often fail to recognize pythiosis, and this results in delayed diagnosis and contributes to the high mortality. Antifungal drugs are ineffective against *P*. *insidiosum*. Approximately, 80% of patients undergo surgical removal of the infected organ (leg or eye). In many advanced cases, surgery fails to eradicate the organism, and ~40% of the patient with vascular pythiosis die from the disease. Better understanding of the biology and pathogenesis of *P*. *insidiosum* could lead to discovery of new methods for prevention, diagnosis, and treatment of pythiosis.

Many pathogenic microorganisms secrete proteins that promote infection by interfering with host cell function and altering host responses [14–22]. For example, the bacterium *Helicobacter pyroli* secretes CagA to perturb a host cell signaling pathway, and leads to development of peptic ulcer [17,18]. The malarial parasite *Plasmodium falciparum* secretes some histidine-rich proteins that facilitate its survival inside red blood cells [19]. In many plant-pathogenic oomycetes, the multifunctional elicitin molecules facilitate infection by triggering host tissue necrosis [22]. The elicitin can also be recognized as a pathogen-associated molecular pattern by plant cells [23–26], and serve as a sterol-carrying protein for acquiring exogenous sterols [27–33].

Recent transcriptome analyses revealed that *P*. *insidiosum* harbors an extensive repertoire of elicitin-domain-containing proteins (~300 proteins), many of which (~60 proteins) are predicted to be secreted [34,35]. The biological role of elicitin in human hosts is unknown. The *P*. *insidiosum* elicitin-encoding gene, *ELI025*, is highly expressed and 5-fold up-regulated when *P*. *insidiosum* hyphae is grown at body temperature (37°C), compared to hyphae grown at room temperature (28°C) [34,35], suggesting that *ELI025* may be required for survival of *P*. *insidiosum* inside a human host. The current study reports on the cloning and expression of *ELI025* for genetic, biochemical and immunological analyses. Molecular characterization of elicitin is a significant step in exploring the biology and virulence of this understudied microorganism and could lead to new strategies for infection control.

## Materials and Methods

### Ethics statement

This study was approved, without requiring informed consent from patients, by the Committee on Human Rights Related to Research Involving Human Subjects, at the Faculty of Medicine, Ramathibodi Hospital, Mahidol University (approval number MURA2012/504S1). An informed consent was not obtained from patients (from whom microorganisms, tissues, and blood samples were obtained) because the data were analyzed anonymously.

### Microorganisms

The *P*. *insidiosum* strains Pi-S, MCC18, and P01, were obtained from a collection of microorganisms that were isolated from clinical samples received for routinely culture identification. All strains were maintained on Sabouraud dextrose agar at room temperature and sub-cultured once a month.

### Serum samples

Three serum samples were obtained from pythiosis patients diagnosed by culture identification or serological tests [36–41]. To serve as controls, three serum samples were obtained from healthy blood donors who came to the Blood Bank Division, Department of Pathology, Ramathibodi Hospital. Rabbit anti-rELI025 sera were purchased from the Biomedical Technology Research Laboratory, Faculty of Associated Medicine, Chiang Mai University, Thailand. To block the rabbit anti-rELI025 antibodies from the rabbit serum, 20 μl of rELI025 (2.4 mg/ml) and 1.5 ml of diluted rabbit serum [1:2,000 in 5% skim milk in TBS (pH 7.5)] were co-incubated with gentle agitation at 4°C overnight. All sera were kept at -20°C until use.

### Protein preparation

Crude protein extracts of *P*. *insidiosum*, including soluble antigen from broken hyphae (SABH; containing intracellular proteins) and culture filtrate antigen (CFA; containing secreted proteins), were prepared according to the methods described by Chareonsirisuthigul et al [41]. Briefly, 100 ml Sabouraud dextrose broth was inoculated from an actively growing *P*. *insidiosum* colony and incubated, with shaking (~150 rpm), at 37°C for 10 days. The organism was killed with 0.02% Thimerosol (Sigma). Hyphae were collected by filtration on a 0.22-μm-pore-size membrane (Durapore, Merck Millipore), and ground in a mortar with pre-cooled distilled water (1.5 g hyphae per 30 ml water). Supernatant, following centrifugation (10,000 x g) of the cell lysate at 4°C for 30 min, was filtered through a 0.22-μm-pore-size membrane (Durapore, Merck Millipore). Both filtered supernatant (SABH) and cell-free broth (CFA) were 100-fold concentrated by ultrafiltration (10,000 molecular weight cut-off membrane; Amicon Ultra 15M, Merck Millipore). Protein concentration was measured by Bradford’s assay [42]. SABH and CFA were stored at -20°C until use.

### Genomic DNA extraction

*P*. *insidiosum* genomic DNA (gDNA) was extracted using the modified method of Lohnoo et al [43]. Briefly, hyphal mat (~500 mg) was transferred to a 2-ml tube containing glass beads (~1,000-μm diameter; Sigma) and 400 μl of homogenizing buffer [0.4 M NaCl, 10 mM Tris—HCl (pH 8.0), 2 mM EDTA (pH 8.0)]. The tube was shaken at 30 Hz for 2 min, in a tissue homogenizer (TissueLyzer, QIAGEN), before adding 20% sodium dodecyl sulfate (final concentration, 2%) and proteinase K (final concentration, 400 μg/ml). The cell lysate was incubated, with gentle inversion, at 55°C, overnight. The sample was then mixed with 300 μl of 6 M NaCl, vigorously vortexed for 30 s, and centrifuged (10,000 x g) at room temperature for 30 min. The supernatant was then mixed with an equal volume of isopropanol, incubated at -20°C for 1 hr, and centrifuged (12,000 x g) at 4°C for 20 min. The gDNA pellet was collected and washed with 70% ethanol, air dried, and resuspended in sterile water. All extracted gDNAs were kept at -20°C until use.

### Plasmid construction

**Fig 1:**
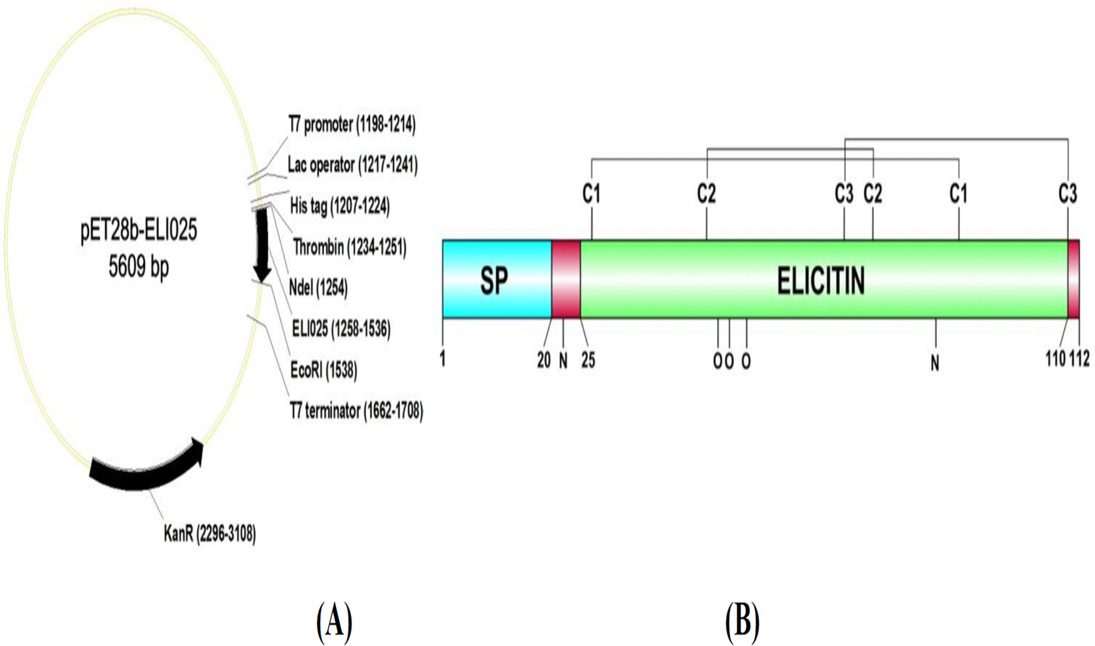
Cloning and expression of *ELI025*. (**A**) Plasmid DNA map of pET28b-ELI025 shows the cloning sites (*Nde-*I and *EcoR-*I) of *ELI025*. Expression of *ELI025* is under the control of the T7 promoter. The numbers in parentheses indicate a location of each plasmid component; (**B**) Protein structure of ELI025 shows a signal peptide (SP; amino acid position 1–20), an elicitin domain (amino acid position 25–110), three disulfide bonds (C1, cysteine position 27 and 91; C2, cysteine position 47 and 76; C3, cysteine position 71 and 110), two predicted N-linked glycosylation sties (N; amino acid position, 22 and 87), and three predicted O-linked glycosylation sties (O; amino acid position 49, 51, and 54).

The full-length ELI025-encoding sequence (NCBI accession number: HS975204) was amplified from the pCR4-blunt-TOPO vector harboring PinsEST#025 cDNA [34], in a 50-μl PCR reaction containing 1.5 μl of PCR product, 1 μl of the Elongase and its buffer mixture (buffer A:B ratio = 1:4) (Invitrogen), 200 μM of dNTPs, and 0.4 μM each of the primer ELI025_NdeI (5’-GGCATCACATATGtacaacgagaccaagccg-3’) and ELI025_EcoRI (5’-CAAGAATTCCTAGGCCTTGCAGCTCGTC-3’). The reaction was carried out in a MyCycler (Biorad) with the following conditions: initial denaturation at 94°C for 30 s, 35 cycles of denaturation at 94°C for 30 s, annealing at 60°C for 30 s, and extension at 68°C for 1.10 min, and final extension at 68°C for 5 min. The PCR product was double digested with *NdeI* and *EcoRI* (New England Biolabs), and directionally cloned into pET28b (Novagen), yielding an in-frame His-tag fusion on the N-terminus of ELI025. The resulting plasmid, pET28b-ELI025 (Fig. 1A), was propagated in the *Escherichia coli* strain DH5α. The sequence of the ELI025-coding region of the plasmid was confirmed using primers, T7-promoter (5’-TAATACGACTCACTATAGGG-3’) and T7-terminator (5’-GCTAGTTATTGCTCAGCGG-3’).

### Protein expression and purification

The recombinant ELI025 protein (rELI025; plasmid pET28b-ELI025) was expressed from the *E*. *coli* strain rosetta-gami2 (DE3) (Novagen). A clone harboring pET28b-ELI025 was grown in the Terrific broth [44], supplemented with tetracycline (12.5 μg/ml), chloramphenicol (34 μg/ml), and kanamycin (30 μg/ml), until the cells reached 0.5 optical density. IPTG (final concentration, 1 mM; Omnipur) was added, before further shaking incubation (250 rpm) at 25°C for 12 hr. The culture was centrifuged (6000 x g) at 4°C for 10 min and the pellet was resuspended in binding buffer [20 mM sodium phosphate buffer (pH 7.4) and 0.1 M NaCl] (1 g pellet per 5 ml binding buffer), mixed with lysozyme (final concentration, 1 mg/ml; BioBasics), incubated on ice for 30 min, sonicated (setting: 50% amplitude, 20 cycles, 10/10-second pulse on/off), and centrifuged (10,000 x g) at 4°C for 30 min. The resulting supernatant was applied to a HiTrap IMAC FF column (GE healthcare), pre-charged with 0.1 M NiCl_2_. The column was sequentially washed with the binding buffer containing 60 and 100 mM imidazole. Protein was eluted from the column with binding buffer containing 500 mM imidazole. The concentration of the purified recombinant protein was determined by Bradford’s assay [42], and kept at -30°C until use.

### SDS-PAGE and Western blot

SABH, CFA, and rELI025 were separated by SDS-PAGE (4% stacking and 12% separating gel) at 150 V, for 65 min, using the Mini-PROTEAN II apparatus (Biorad). Proteins were stained with either Coomassie blue R-250 or Silver staining kit (Thermo Scientific). The Image Lab 3.0 program (Biorad) was used to estimate protein molecular weight (kilo Dalton; kDa) based on migration of pre-stained broad range protein markers (Biorad). For Western blot analysis, the separated proteins were transferred and immobilized onto a 0.2-μm-pore-size PVDF membrane (Merck Millipore), using the Biorad Mini Trans-Blot cell (setting: 100 V for 60 min). The blotted membrane was blocked with 5% skim milk (Sigma) in blocking buffer [TBS; 150 mM NaCl, 10 mM Tris-HCl (pH 7.5)] at 4°C, overnight, or room temperature for an hour. The membrane was washed 3 times with the washing buffer [TTBS; 500 mM NaCl, 20 mM Tris-Cl, and 0.1% Tween-20 (pH 7.5)]. The membranes were incubated with the primary antibodies diluted in the blocking buffer [1:1,000 for mouse anti-6x histidine antibody (Merck Millipore); 1:2,000 for rabbit anti-rELI025 serum; 1:1,000 for patient serum] for 2 hr at room temperature (anti-6x histidine and anti-rELI025) or overnight at 4°C (patient serum), and washed 3 times with TTBS. The secondary antibodies, diluted in the blocking buffer [1:2,000 for goat anti-mouse IgG conjugated with horseradish peroxidase (Merck Millipore); 1:5000 for goat anti-rabbit IgG conjugated with alkaline phosphatase (Southern Biotech); 1:3000 for goat anti-human IgG conjugated with horseradish peroxidase (Biorad)], were added to the membrane and incubated for 2–3 hr at room temperature. After washing the membrane 3 times with TTBS, substrate and chromogen (4CN and H_2_O_2_ for horseradish peroxidase; NBT and BCIP for alkaline phosphatase) were added to develop Western blot signals. Intensities of Western blot bands were quantitated by the GelQuant.NET software (http://biochemlabsolutions.com/GelQuantNET.html).

### Mass spectrometric analysis

The 10- and 15-kDa bands present on SDS-PAGE gel and Western blot were excised from gel and PVDF membrane, respectively. Proteins were extracted and trypsin digested, using the method described by Shevchenko et al. [45]. The digested proteins were analyzed by an Ultimate 3000 LC System (Dionex, USA) coupled to an HCTultra PTM Discovery System (Bruker Daltonics Ltd., U.K.) at the Proteomics Research Laboratory, Genome Institute, National Center for Genetic Engineering and Biotechnology, Thailand. The Bruker Daltonics Data Analysis version 4.0 (Bruker Daltonics Ltd., U.K.) was used to analyze raw mass spectrometric data. The MASCOT software (Matrix Science, UK) was used to search the obtained MS and MS/MS data against ~15,000 genome-derived predicted proteins of *P*. *insidiosum* (unpublished data).

### Deglycosylation of glycoprotein

A glycoprotein deglycosylation kit (Calbiochem) was used to remove sugar moieties (N- and O-linked glycosylation) from the native ELI025 in CFA. Briefly, 50 μg of CFA, 10 μl of 5x kit deglycosylation buffer, 2.5 μl of the kit denaturation solution, and distilled water were mixed to the final volume of 50 μl. The mixture was heated at 100°C for 5 min and cooled down to room temperature, before adding 2.5 μl of 15% TRITON X-100. To remove N-glycosyl groups, 1 μl of N-glycosidase F was added to the reaction. To remove O-glycosyl groups, 1 μl of the enzyme mixture, including α-2–3,6,8,9-neuraminidase, endo-α-N-acetylgalactosaminidase, β1,4-galactosidase, and β-N-acetylglucosaminidase, was added to the reaction. The protein-enzyme mixture was incubated at 37°C for 3 hr.

### Immunohistochemical staining assay

Immunohistochemical staining assay was performed, using the method described by Keeratijarut *et al* [46], with some modifications. A paraffin-embedded tissue section (4-μm thickness) from a patient with vascular pythiosis was pretreated with xylene and absolute ethanol (Merck), before washing with phosphate buffered saline (PBS; pH 7.4). The tissue section was incubated with Tris-EDTA buffer (TE buffer; pH 9.0) at 95°C for 40 min, treated with 10% H_2_O_2_ in PBS for 10 min, and washed with PBS. Then, the tissue section was incubated with 200 μl of either rabbit pre-immune serum or rabbit anti-rELI025 serum (1:16,000 in PBS) at 4°C overnight, washed with PBS (5 min each), and incubated with 200 μl of mouse anti-rabbit IgG antibody conjugated with horseradish-peroxidase (Thermo Scientific, USA) for 30 min. To develop color, the substrate 3,3’-diaminobenzidine tetrahydrochloride (DAKO, USA) was added to the tissue section and incubated at room temperature for 5 min. The tissue section was counterstained with hematoxylin before examination with a light microscope (ECLIPSE Ci, Nikon, Japan).

### Polymerase chain reaction and DNA sequencing

A draft genome sequence of the *P*. *insidiosum* strain Pi-S (unpublished data) was used to design primers for PCR amplification of the rELI025-encoding sequence and its promoter region (ELI025_promoter_F2, 5’-CATGGACAGCGTCATCTCTGG-3’; ELI025_promoter_R1, 5’-GCGTCAAGATGAGAAACGAGG-3’). Each amplification reaction was performed in a 50-μl reaction containing 100 ng of genomic DNA template, 0.02 U/μl of DNA polymerase (Phusion), 1x Phusion buffer, 200 μM of dNTPs, and 0.5 μM each of the primer. The amplification was carried out in a Mastercycler Nexus thermal cycler (Eppendorf), with the following conditions: an initial denaturation at 98°C for 30 s, 35 cycles of denaturation at 98°C for 10 s, annealing at 55°C for 30 s, and extension at 72°C for 1 min, and a final extension at 72°C for 10 min. The PCR products were purified using a NucleoSpin Gel and PCR Clean-up kit (Macherey-Nagel) and assessed for amount and size by 1% gel electrophoresis.

Direct sequencing of PCR products was performed using a BigDye terminator V3.1 cycle sequencing kit (Applied Biosystems) and an ABI Prism 3100 Genetic Analyzer (Applied Biosystems). The primers used for sequencing included ELI025_F1 (5’-TACAACGAGACCAAGCCGTG-3’), ELI025_R1 (5’-GGCCTTGCAGCTCGTCTC-3’), ELI025_promoter_F2, ELI025_promoter_R1, ELI025_promoter_seqF1 (5’-CGCCCCCTTTCTTCCCGAC-3’) and ELI025_promoter_seqR1 (5’-CCAACCAGACGCCGTCTG-3’). The sequences were analyzed using the ABI Prism DNA Sequencing Analysis software (Applied Biosystems, USA).

### Bioinformatic analysis

The molecular weight of ELI025 was calculated by ProtParam [47]. Signal peptide, transmembrane domain, N- and O-linked glycosylation and GPI-anchor of ELI025 were predicted using the SignalIP program version 4.0 [48], the TMHMM program version [49], the NetNGlyc (http://www.cbs.dtu.dk/services/NetNGlyc), and NetOGlyc [50] programs, and the big-PI predictor [51], respectively. The promoter and ELI025-coding sequences from all *P*. *insidiosum* strains used in this study were aligned and compared using the ClustalX program version 2 and the GeneDoc program [52,53].

### Homologous protein search

The elicitin domain sequence of ELI025 was used to BLAST search for elicitin homologous proteins encoded in the genomes and transcriptomes, or present in the proteomes of 18 oomycetes, 10 fungi, 4 algae, 3 diatoms, and one protozoan [54–72] (Table 1). The cut-off E-value for BLAST searches was ≤ 1 x 10^-4^. If a BLAST search of particular genome database was not possible online, then a local BLASTP and TBLASTN search was performed using the BLAST 2.2.28+ program (http://www.ncbi.nlm.nih.gov/news/04-05-2013-blast-2-2-28/).

**Table 1:** BLAST search of the ELI025 amino acid sequence against the genomes, transcriptomes, or proteomes of 18 oomycetes, 10 fungi, 4 algae, 3 diatoms, and one protozoan (the cut-off E-value ≤ 1 x 10–4).

Organisms	Group	Subgroup	Number of BLAST hits	E-value of the best BLAST hit	References
*Pythium ultimum*	Oomycete	Pythiales	27	3.30E-31	[54]
*Pythium aphanidermatum*	Oomycete	Pythiales	20	1.00E-15	[54]
*Pythium irregulare*	Oomycete	Pythiales	18	2.00E-34	[54]
*Pythium arrhenomanes*	Oomycete	Pythiales	17	8.80E-16	[54]
*Pythium iwayamai*	Oomycete	Pythiales	14	7.00E-30	[54]
*Pythium vexans*	Oomycete	Pythiales	14	1.00E-20	[54]
*Phytophthora sojae*	Oomycete	Peronosporales	26	1.46E-22	[55]
*Phytophthora ramorum*	Oomycete	Peronosporales	25	1.00E-21	[55]
*Phytophthora parasitica*	Oomycete	Peronosporales	16	1.11E-14	BI^a^
*Phytophthora capsici*	Oomycete	Peronosporales	15	7.02E-20	[56]
*Phytophthora cinnamomi*	Oomycete	Peronosporales	14	5.51E-20	JGI^b^
*Phytophthora infestans*	Oomycete	Peronosporales	10	2.11E-16	BI^a^
*Pseudoperonospora cubensis*	Oomycete	Peronosporales	6	2.00E-07	[57]
*Hyaloperonospora arabidopsis*	Oomycete	Peronosporales	2	1.00E-06	[58]
*Albugo laibachii*	Oomycete	Albuginales	1	6.40E-13	[59]
*Aphanomyces euteiches*	Oomycete	Saprolegniales	-	-	[60]
*Saprolegnia diclina*	Oomycete	Saprolegniales	-	-	BI^a^
*Saprolegnia parasitica*	Oomycete	Saprolegniales	-	-	[61]
*Phaeodactylum tricornutum*	Diatom	Bacillariophyta	-	-	[62]
*Pseudo-nitzschia multiseries*	Diatom	Bacillariophyta	-	-	JGI^b^
*Thalassiosira pseudonana*	Diatom	Bacillariophyta	-	-	[63]
*Aurantiochytrium limacinum*	Microalgae	Labyrinthulida	-	-	JGI^b^
*Nannochloropsis gaditana*	Microalgae	Eustigmatophyceae	-	-	[64]
*Aureococcus anophagefferens*	Brown tide algae	Pelagophyceae	-	-	[65]
*Ectocarpus siliculosus*	Brown algae	PX clade	-	-	[66]
*Blastocystis hominis*	Protozoan	Blastocystis	-	-	[67]
*Aspergillus* spp.	Fungi	Ascomycota	-	-	[68]
*Candida* spp.	Fungi	Ascomycota	-	-	[69]
*Fusarium oxysporum*	Fungi	Ascomycota	-	-	BI^a^
*Histoplasma capsulatum*	Fungi	Ascomycota	-	-	BI^a^
*Paracoccidioides brasiliensis*	Fungi	Ascomycota	-	-	BI^a^
*Pneumocystis jirovecii*	Fungi	Ascomycota	-	-	[70]
*Mucor circinelloides*	Fungi	Zygomycota	-	-	BI^a^
*Rhizopus delemar*	Fungi	Zygomycota	-	-	BI^a^
*Rhizopus oryzae*	Fungi	Zygomycota	-	-	[71]
*Cryptococcus neoformans*	Fungi	Basidiomycota	-	-	[72]

^a^ Broad institute genome database

^b^ Genome portal of the Department of Energy Joint Genome Institute

### Phylogenetic analysis

Elicitin domain sequences from different oomycete organisms (Table 1) were analyzed online at http://www.phylogeny.fr/ [73]. The sequences were aligned by MUSCLE [74], and phylogenetic relationships were calculated by Neighbor-joining with 1,000 bootstraps [75] and the Jones-Taylor-Thornton matrix substitution model [76]. A phylogenetic tree was generated by TreeDyn [77].

### Nucleotide sequence accession numbers

All ELI025-coding sequences from *P*. *insidiosum* strain Pi-S, MCC18, and P01 have been submitted to the DNA data bank of Japan (DDBJ), under accession numbers AB971191 to AB971193, respectively.

## Results

### Structures of *ELI025* and its gene product

The DNA sequence covering the 5’-untranslated region, coding sequence, and 3’-untranslated region of the *ELI025* gene was successfully PCR-amplified from gDNA of three different *P*. *insidiosum* strains: Pi-S (1,106-bp long; accession number, AB971191), MCC18 (1,056-bp long; accession number, AB971192), and P01 (1,036-bp long; accession number, AB971193). No intron was identified when the gDNA-derived (accession number, AB971191–3) and mRNA-derived (accession number, HS975204 and FX528334) ELI025-coding sequences were aligned. Analyses of the coding sequences for the *ELI025* alleles of three different strains of *P*. *insidiosum* by ClustalX version 2 [52] and GeneDoc [53] programs showed 98–99% identity and 99–100% similarity with each other (data not shown).

**Fig 2:**
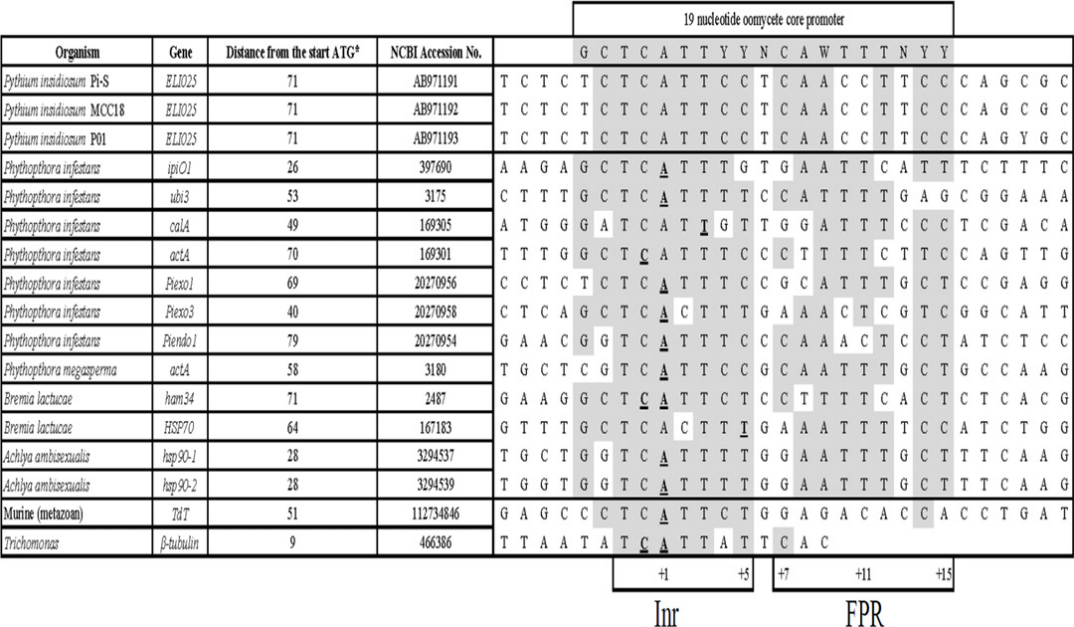
Sequence alignment of core promoter regions of the *P. insidiosum ELI025* gene and various genes from several oomycetes and parasites. The *ELI025* sequences (accession number AB971191–3), used for the alignment, are derived from three different *P*. *insidiosum* strains. Conserved nucleotides are highlighted in grey. The underlined letters indicate the known transcriptional start site, and is indicated below as "+1". Two putative core promoter components, an initiator element (Inr; 5’-TCATTCC-3’; positions-2 to +5) and a flanking promoter region (FPR; 5’-CAACCTTCC-3’; positions +7 to +15), are found in the upstream region of all genes. (Abbreviation: NCBI, National Center for Biotechnology Information).

The 5'-untranslated and -flanking DNA sequences of the *ELI025* gene from the three *P*. *insidiosum* strains were compared with that of various genes from several oomycetes and parasites (Fig. 2). These sequences share a 19-nucleotide oomycete core-promoter sequence, located between 9 and 79 nucleotides upstream of the start codon (Fig. 2). Two putative core-promoter components, an initiator element (Inr; 5’-TCATTCC-3’) and a flanking promoter region (FPR; 5’-CAACCTTCC-3’), were identified in this region of *ELI025* (Fig. 2). A predicted transcription start site (+1; Fig. 2) of the *ELI025* gene is within the Inr element. A TATA box was not observed in the promoter region of *ELI025*.

The predicted full-length ELI025 protein sequences (112 amino acids long) from the three *P*. *insidiosum* strains were 100% identical. A predicted signal peptide of ELI025 covered the first 20 N-terminal amino acids (Fig. 1B). The calculated molecular weights of ELI025, with and without the signal peptide, were 12 and 10 kDa, respectively. The elicitin domain spanned from amino acid position 25 to 110. By analogy to known elicitins [34], three disulfide bonds (Cys27/Cys91, Cys47/Cys76, and Cys71/Cys110) were identified within the elicitin domain of ELI025 (C1, C2, C3; Fig. 1B). There are two predicted N-linked glycosylation sites at amino acid positions 22 and 87, and three predicted O-linked glycosylation sites at amino acid positions 49, 51, and 54 (Fig. 1B). Neither a GPI anchor nor a transmembrane region are predicted for ELI025.

### Homologous proteins of ELI025

**Fig 3:**
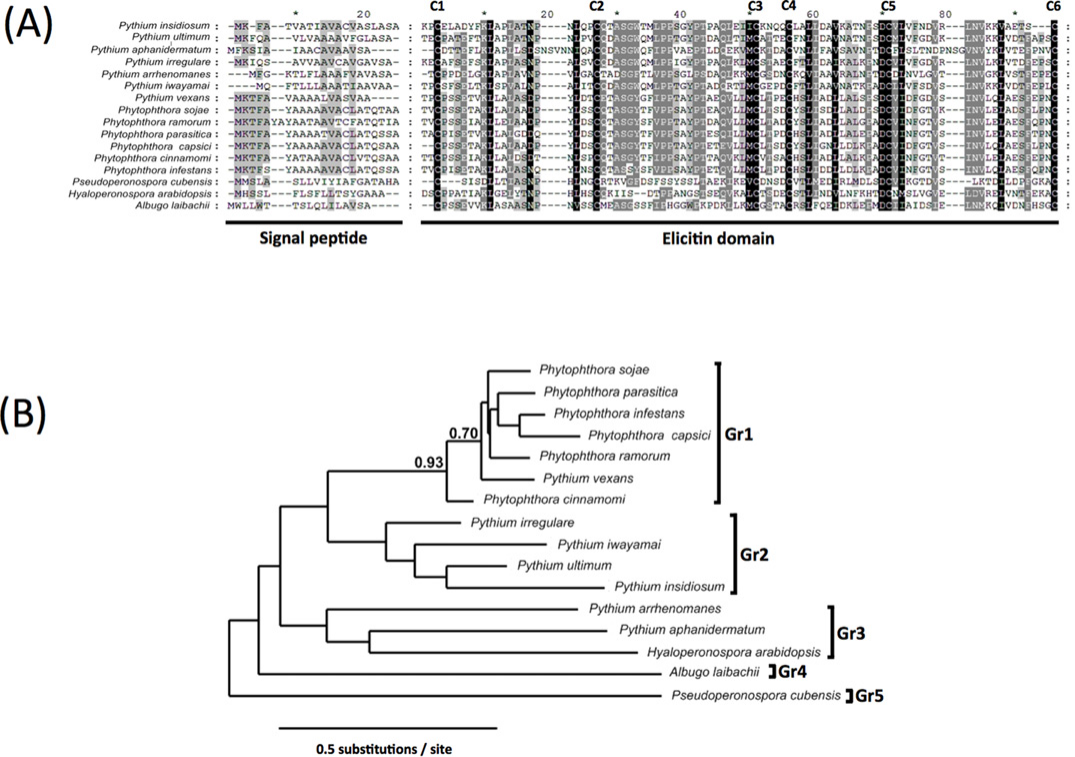
Sequence alignment and phylogenetic analysis of elicitin proteins. (**A**) Signal peptide (length, 17–23 amino acids) and elicitin domain (length, 83–94 amino acids) sequences of *P*. *insidiosum* ELI025 and the top BLAST hit proteins of 15 other oomycetes (Table 1) were aligned and compared. C1–C6 indicate conserved cysteine residues; (**B**) Phylogenetic analysis of elicitins by the neighbor-joining method. The phylogenetic tree, constructed from elicitin domain sequences of *P*. *insidiosum* ELI025 and the top BLAST hit proteins of 15 other oomycetes (Table 1), shows three major clades (as indicated by Gr1, Gr2 and Gr3; containing multiple sequences per clade) and two minor clades (as indicated by Gr4 and Gr5; containing one sequence per clade). Only the branch support values of 70% or more are shown at corresponding nodes.

The ELI025 amino acid sequence was used for BLAST analyses of the genomes, transcriptomes, and proteomes of 36 different microorganisms (Table 1). No significant BLAST hit was identified in non-oomycete organisms. Three oomycetes (*A*. *euteiches*, *S*. *diclina*, and *S*. *parasitica*), which belong to the subgroup Saprolegniales, lacked sequences homologous to ELI025. A number of BLAST hits were found in 15 species of oomycetes, including *Pythium* spp. (14–27 hits), *Phytophthora* spp. (10–26 hits), *P*. *cubensis* (6 hits), *H*. *arabidopsis* (2 hits), and *A*. *laibachii* (1 hit). A signal peptide and an elicitin domain were identified in these top BLAST hit proteins (Fig. 3A). The similarity between the ELI025 signal peptide and signal peptides of the other oomycetes’ elicitins (17–23 amino acids long) was high (mean, 44%; median, 47%; range, 19–71%). Elicitin domain sequences of ELI025 and the other top BLAST hit proteins (83–94 amino acids long) contained 6 conserved cysteine residues (Fig. 3A). Phylogenetic analysis, based on the elicitin domain sequences, divided the oomycetes into 5 closely related groups (Gr1–5): Gr1 contained all *Phytophthora* species; Gr2 and Gr3 comprised mainly *Pythium* species; Gr4 included *A*. *laibachii* and Gr5 had only *P*. *cubensis* (Fig. 3B).

### ELI025 is a major secreted non-immunogenic glycoprotein

**Fig 4:**
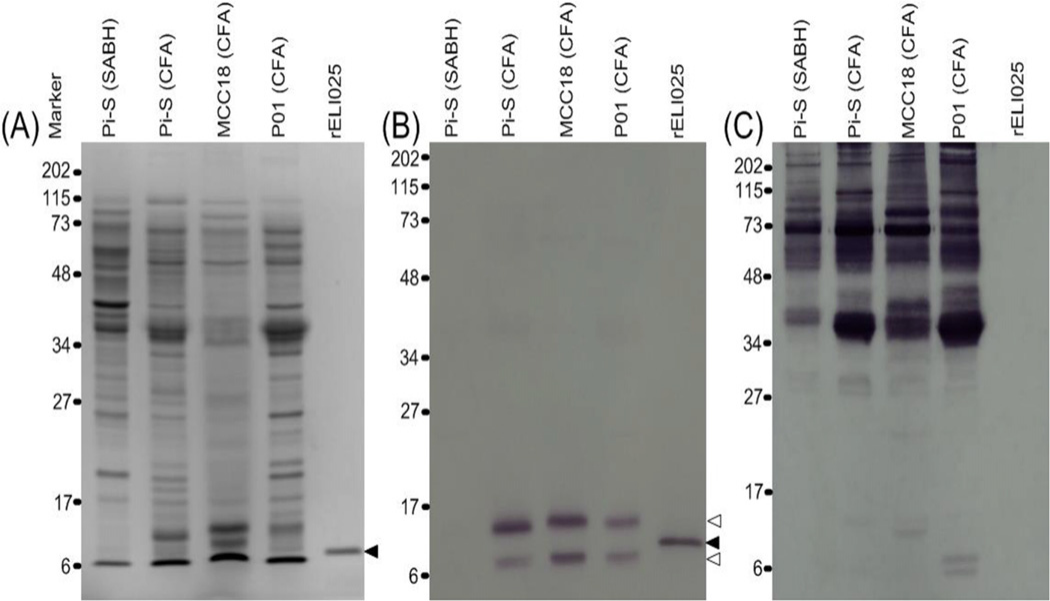
Immunoreactivity of the recombinant protein rELI025 and crude protein extracts of *P. insidiosum*. Crude proteins (i.e., SABH and CFA) extracted from three different strains of *P*. *insidiosum* (Pi-S, MCC18, and P01) and rELI025 are separated in a SDS-PAGE gel **(A)**. The separated proteins are analyzed by Western blot, using the rabbit anti-rELI025 antibodies **(B)**, or sera from patients with pythiosis **(C)**, as probe. The black arrow head indicates the 12.4 kDa band of rELI025. The white arrow heads indicate the 10- and 15-kDa bands of native ELI025. The numbers represent protein molecular weights standards, in kDa. (Abbreviations: SDS-PAGE, Sodium dodecyl sulfate polyacrylamide gel electrophoresis; CFA, culture filtrate antigen; SABH, soluble antigen from broken hyphae; rELI025, recombinant ELI025).

The recombinant protein, rELI025, was successfully expressed and purified from *E*. *coli* (protein yield: 2 mg per 1 liter of bacterial culture). Purity of rELI025 was at least 99%, as demonstrated by silver staining analysis of SDS-PAGE gel. The molecular weight of rELI025 in the SDS-PAGE gel was estimated to be 12.4 kDa (Fig. 4A). rELI025 appeared as an intense 12.4-kDa Western blot band, when reacted with the mouse anti-6x histidine-tag antibody (data not shown) or the rabbit anti-rELI025 antibodies (Fig. 4B).

Gel-separated total proteins from crude extracts or supernatants of three *P*. *insidiosum* strains, had molecular weights ranging from 6 to 115 kDa (Fig. 4A). In Western Blots, rabbit anti-rELI025 serum reacted only with the 10- and 15-kDa bands in CFA (culture filtrate antigen), which contains secreted proteins of *P*. *insidiosum* (Fig. 4B). The rabbit anti-rELI025 serum did not react any proteins in SABH (soluble antigens from broken hyphae), which contains intracellular proteins (Fig. 4B). The rabbit pre-immune serum did not detect any proteins in SABH or CFA. If the rabbit anti-rELI025 serum is pre-absorbed with rELI025 protein prior to Western Blot detection, the band intensities for the 10- and 15-kDa proteins were reduced by ~85% (data not shown).

**Fig 5:**
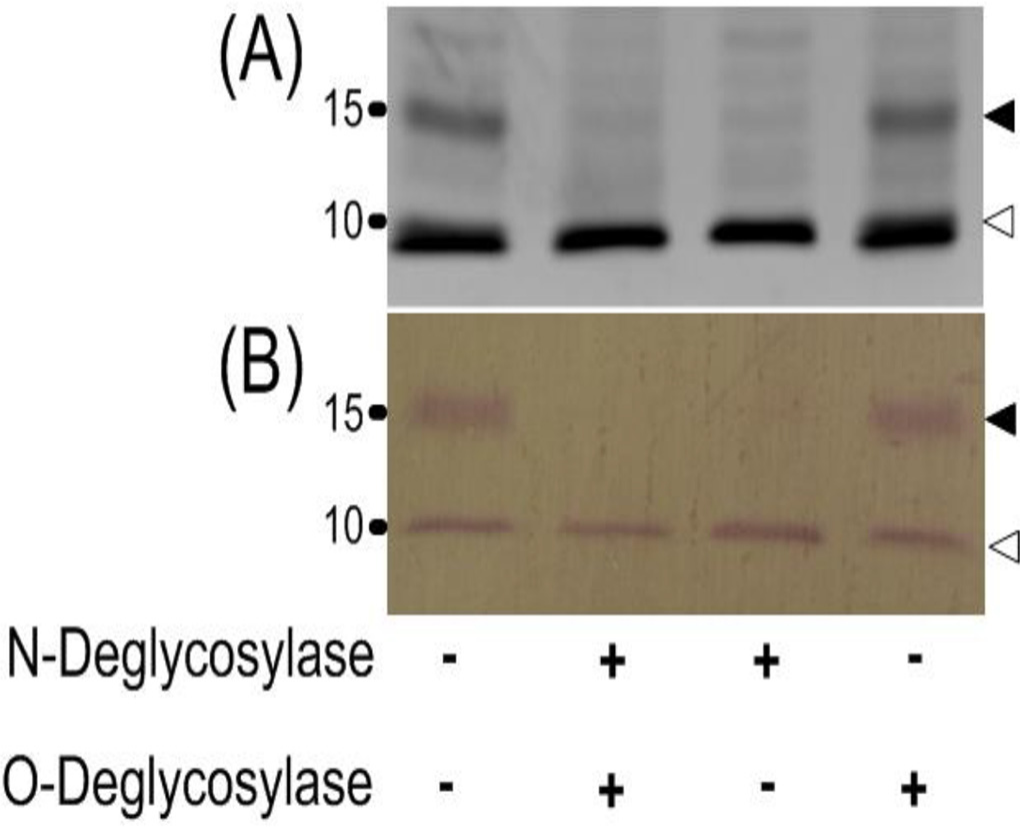
Protein deglycosylation of ELI025. CFA proteins were untreated (control; Lane 1) or treated with either N-Deglycosylase (N-glycosidase F; Lane 3), or O-Deglycosylase (a mixture of α-2–3,6,8,9-neuraminidase, endo-α-N-acetylgalactosaminidase, β-1,4-galactosidase, and β-N-acetylglucosaminidase; Lane 4), or both N- and O-Deglycosylases (Lane 2). The enzyme-treated proteins were separated on a SDS-PAGE gel **(A)**, and were further analyzed by Western blot, using the rabbit anti-rELI025 antibodies as primary antibody **(B)**. Only the low molecular weight portion of the gel and blot are shown. The black and white arrow heads point out the 15-kDa and 10-kDa bands. (Abbreviations: SDS-PAGE, Sodium dodecyl sulfate polyacrylamide gel electrophoresis; CFA, culture filtrateantigen; rELI025, recombinant ELI025).

The native ELI025 (nELI025) in CFA was treated with protein deglycosylases to remove either N- or O-linked glycosyl adducts. The SDS-PAGE and Western blot profiles (probed with the rabbit anti-rELI025 serum) show that the 15-kDa band disappears in the CFA treated with the N-linked deglycosylation enzyme (either alone or in combination with the O-linked deglycosylase; Fig. 5). In contrast, the 10- and 15-kDa bands were both present in CFA treated with O-linked deglycosylase or in the no enzyme control.

**Fig 6:**
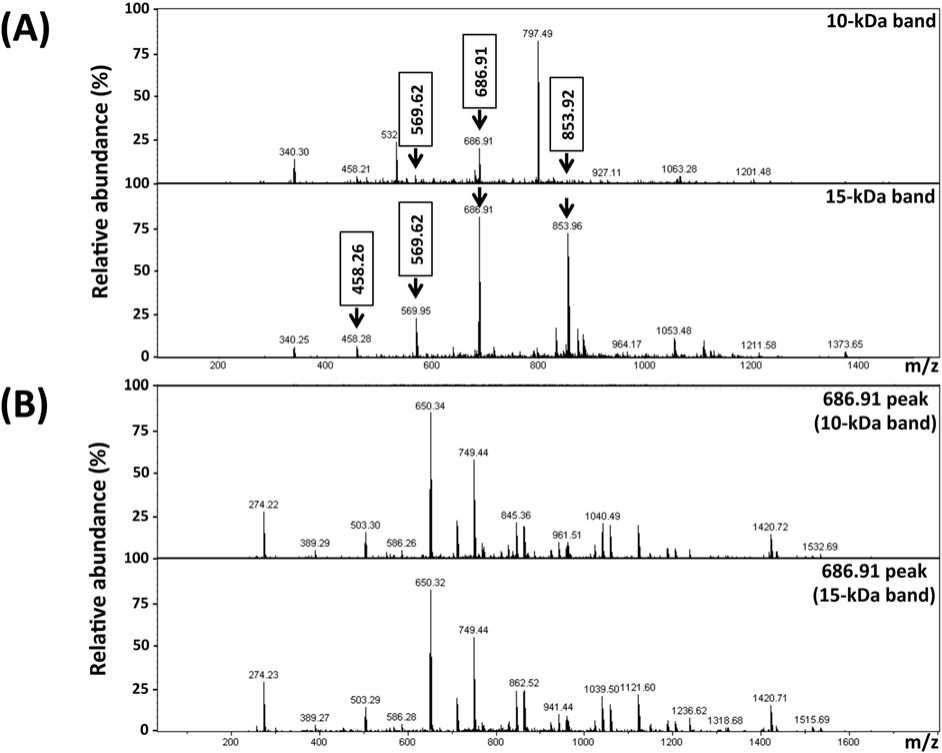
Mass spectrometric analyses of ELI025 by LC-MS/MS. (**A**) MS spectra of the 10- and 15-kDa SDS-PAGE band-derived proteins. The arrows indicate peptides with the mass-to-charge ratio (m/z), including 458.3, 569.6, 686.9 and 853.9, that match the ELI025 protein. Peptide sequences corresponding to the ELI025-matched peaks are shown in Table 2; (**B**) MS/MS spectra of the 686.91 peaks from the 10- and 15-kDa SDS-PAGE band-derived proteins.

The 10- and 15-kDa bands excised from SDS-PAGE gel (Fig. 3A) and Western blot membrane (Fig. 3B) were analyzed by LC-MS/MS (see Methods). MASCOT analysis of MS data showed that the 10-kDa SDS-PAGE band-derived peptides with mass-to-charge ratio (m/z) of 569.6, 686.9 and 853.9, and the 15-kDa SDS-PAGE band-derived peptides with m/z of 458.3, 569.6, 686.9 and 853.9, matched the ELI025 protein in the *P*. *insidiosum*’s proteome (Fig. 6A; Table 2). No peptide mass of the 10- and 15-kDa band excised from Western blots matched ELI025. Further MASCOT analyses of MS/MS data of the 10- and 15-kDa SDS-PAGE band-derived 686.9 peak, showed that the corresponding peptides had nearly-identical spectra (Fig. 6B), and matched the same peptide sequence (KNQQCLALLDAVKA) predicted for ELI025. Similarly, MASCOT analyses of MS/MS data of the 10- and 15-kDa SDS-PAGE band-derived 853.9 peak, revealed that the corresponding peptides had nearly-identical spectra (data not shown), and matched another peptide sequence (KATNPSDCVLVFNDVRL) predicted for ELI025.

**Table 2:** Mass spectrometric analyses of the 10- and 15-kDa SDS-PAGE band-derived proteins showing mass-to-charge ratio (m/z), average mass of peptide (Mr; calculated by MASCOT software), peptide sequences (identified by MASCOT software), BLAST search result (against ~15,000 genome-derived predicted proteins of P. insidiosum), and amino acid position of identified peptides.

SDS-PAGE band	m/z	M_r_	Peptide sequence	BLAST search result	Amino acid position
10-kDa	569.62	1705.81	KATNPSDCVLVFNDVRL	ELI025	84–100
10-kDa	686.91	1371.72	KNQQCLALLDAVKA	ELI025	72–85
10-kDa	853.92	1705.81	KATNPSDCVLVFNDVRL	ELI025	84–100
15-kDa	458.26	1371.72	KNQQCLALLDAVKA	ELI025	72–85
15-kDa	569.62	1705.81	KATNPSDCVLVFNDVRL	ELI025	84–100
15-kDa	686.91	1371.72	KNQQCLALLDAVKA	ELI025	72–85
15-kDa	853.92	1705.81	KATNPSDCVLVFNDVRL	ELI025	84–100

Three serum samples each from pythiosis patients and normal individuals (control) were used as primary antibodies in Western blot to detect rELI025 or nELI025 in SABH and CFA. All control sera did not detect any proteins in SABH and CFA (data not shown). While pythiosis sera detected relatively-high molecular weight proteins of SABHs and CFAs (> 30 kDa), they failed to detect many lower molecular weight proteins, including the 10- and 15-kDa (representing nELI025). The patient sera also failed to react with the 12.4-kDa rELI025 band (Fig. 4C).

**Fig 7:**
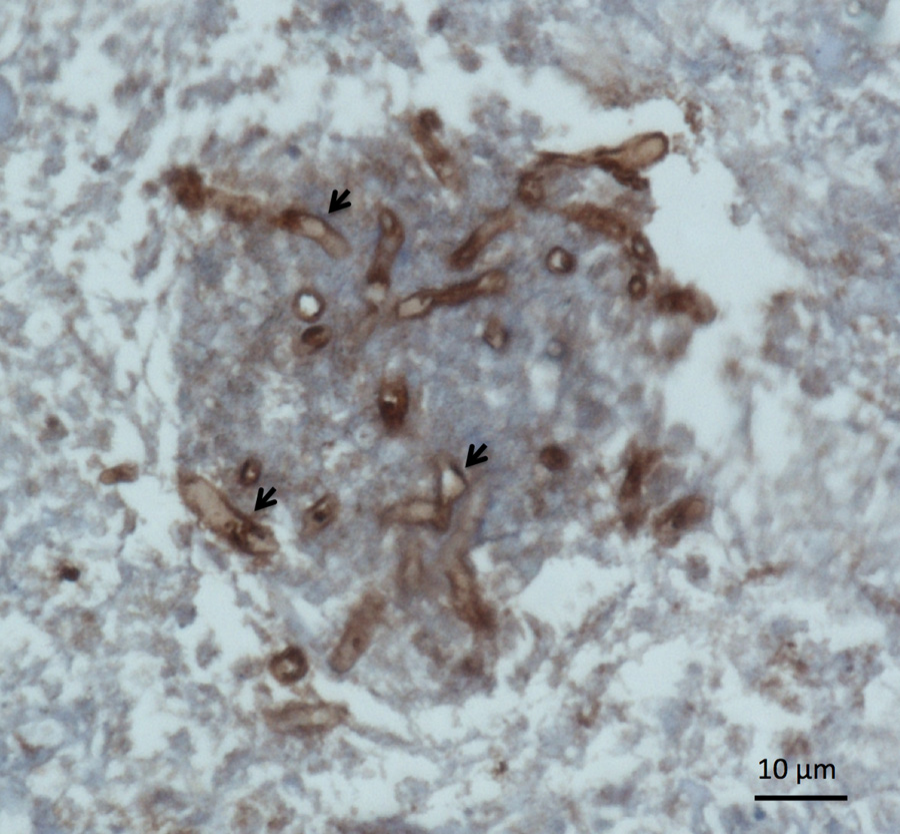
Cellular location of ELI025. Infected arterial tissue from a pythiosis patient was sequentially stained with rabbit anti-rELI025 serum, as the primary antibody, and then mouse anti-rabbit IgG antibody conjugated with horseradish-peroxidase, as the secondary antibody (see Materials and Methods). Images of the hyphae and location of ELI025 (indicated by arrows) were captured with a bright-field microscope. The scale bar represents 10 μm.

An immunohistochemical staining assay, using the rabbit anti-rELI025 serum, was used to target cellular localization of the *P*. *insidiosum* nELI025 in an infected arterial tissue. nELI025 markedly localized at the cell surface and surrounding areas (Fig. 7). No signal was detected with the rabbit pre-immune serum.

## Discussion

The elicitin domain of ELI025 was predicted to contain three disulfide bonds (Fig. 1B), which is a crucial characteristic of elicitin [34]. The *E*. *coli* strain rosetta-gami2 (DE3) was used to express ELI025 based on its reported facility in proper disulfide bond formation. The rabbit anti-rELI025 antibody, detected two proteins in CFA (10- and 15-kDa), but not in SABH (Fig. 4B). The rabbit anti-rELI025 serum, pre-absorbed with rELI025, failed to effectively detect any proteins in CFA, indicating that the rabbit anti-rELI025 antibodies were specific to ELI025. The 10- and 15-kDa bands could be different proteins (i.e., other elicitins) or different isoforms of the same protein (ELI025 contains several predicted glycosylation linkages). Deglycosylation of CFA proteins indicated that the 10-kDa band represents nELI025 without glycosylation, while the 15-kDa band represents nELI025 with predominant N-linked glycosylation (Fig. 5). Thus, nELI025 is a secreted glycoprotein, with two isoforms. The slightly-larger size of rELI025 (12.4 kDa) compared to the non-glycosylated nELI025 (10 kDa) is expected based on its expression in *E*. *coli* as a fusion with Thrombin and a His tag (Fig. 1A).

Mass spectrometric analyses were used to confirm the identity of ELI025 in the 10- and 15-kDa bands of SDS-PAGE gel (Fig. 4A). The sequences, determined by MS and MS/MS analyses, of the peptide mass 686.9 (KNQQCLALLDAVKA) and 853.9 (KATNPSDCVLVFNDVRL) of either the 10- or 15-kDa SDS-PAGE bands matched perfectly with the ELI025 predicted protein sequence. However, no peptide masses matching ELI025 were detected in the 10- and 15-kDa Western blot bands. This may result from the Western blot bands being contaminated with blocking reagent, primary antibody, secondary antibody, enzyme, and substrate, which may compromise detection sensitivity. As an alternative method to determine the identity of the 10- and 15-kDa bands in PVDF membrane, we used rabbit anti-ELI025 antibodies against rELI025 in Western blot analysis. The rabbit anti-rELI025 antibodies reacted only with the 10- and 15-kDa bands (Fig. 4B), suggesting that the protein detected in the Western blots is ELI025.

Until recently, there were few genetic and molecular studies done in *P*. *insidiosum*, and to date, there is no transformation system for introducing foreign or modified *P*. *insidiosum* genes into the organism. In other oomycetes, transformations systems have been developed, and some of these depend on the *hsp70* and *ham34* promoters from the oomycete *Bremia lactucae* for transgene expression [78–81]. Since the upstream region of *ELI025* has conserved sequence found in the core promoter elements of many oomycete genes including *hsp70* and *ham34* (Fig. 2), it may be possible to use already-developed transformation vectors such as pTH210, pHAMT34H, pHAMT35N/SK, and pHAMT35G [79], which utilize the *hsp70* and *ham34* promoters, for developing transformations systems in *P*. *insidiosum*. In addition, since *ELI025* is highly expressed, its upstream region could be used as a driving promoter for DNA transformation in *P*. *insidiosum*.

Elicitins form a unique group of proteins that have been found previously in two oomycete genera (*Phytophthora* spp. and some *Pythium* spp.), but not in fungi or bacteria [22,82,83]. In this study, we performed a similarity search of elicitin domain-containing proteins in the publicly-available genome, transcriptome, and proteome databases of various oomycetes (Table 1). In addition to *Phytophthora* and *Pythium* species, elicitin homologs were also found in oomycete genera *Pseudoperonospora*, *Hyaloperonospora*, and *Albugo* (Table 1). As expected, phylogenetic analysis grouped the top BLAST hit elicitins of these oomycetes according to their genera based on previous classifications: *Phytophthora* species in Gr1 (with an exception for *P*. *vexans*), *Pythium* species in Gr2 and Gr3 (with an exception for *H*. *arabidopsis*), *Albugo* species in Gr4, and *Pseudoperonospora* species in Gr5 (Fig. 3B). The conserved homology of elicitins among the closely related species also extended to both their core promoter sequences (Fig. 2) and their signal sequences (Fig. 3A). It should be noted that elicitins found thus far are in the more closely-related subgroups Pythiales, Peronosporales, and Albuginales. In contrast, no elicitin homologs were detected in *Aphanomyces* and *Saprolegnia* species, which belong to Saprolegnales, a more distantly-related oomycete lineage. This finding suggests that the origin and expansion of elicitins occurred after splitting off the oomycetes from its ancient progenitor and between the Saprolegnales lineage, and the ancestor lineage of the Pythiales, Peronosporales, and Albuginales.

Based on an extensive genome search (Table 1), elicitins are found in many oomycetes, but absent in all non-oomycete organisms, such as, fungi. Thus, elicitins are a signature character of the oomycetes. Among oomycetes, *P*. *insidiosum* is a notorious human pathogen. It shares microscopic features with some pathogenic fungi (such as, *Aspergillus* species, *Fusarium* species, and Zygomycetes). This can lead to misdiagnosis of pythiosis as a fungal infection [46,84], and results in delayed and improper treatment of patients. Because of the uniqueness of the elicitins to *P*. *insidiosum* among human pathogens, detection of *ELI025* or its gene product could aid in the development of more specific diagnostic tests for pythiosis, such as using the anti-rELI025 antibodies to detect *P*. *insidiosum* in infected tissue.

The detection of ELI025 in CFA, together with the predicted amino acid sequence harboring a signal peptide, indicate that ELI025 is a secreted protein. Additionally, the immunohistochemical staining assay of the infected tissue from a pythiosis patient showed localization of ELI025 at *P*. *insidiosum*’s cell surface and surrounding areas (Fig. 7). This evidence suggest that, in *P*. *insidiosum*, ELI025 is expressed and secreted, both during *in vitro* growth and during infection of host tissue. Elicitins, secreted by the plant-pathogenic oomycetes, are beneficial to the pathogens by effecting host response and triggering programed cell death [23,24]. The role of elicitin secreted by *P*. *insidiosum* in humans is unknown. *Pythium* species are thought to be sterol auxotrophic microorganisms [22,32,33]. Like the elicitins from the plant-pathogenic oomycetes, *P*. *insidiosum* ELI025 has been predicted to contain a hydrophobic cavity that can bind a sterol molecule, implying that it can function as a sterol-carrying protein [31,34,85–88]. Western blot assays showed that the small proteins (< 30 kDa) in CFA, including nELI025, were not recognized by sera from patients with pythiosis (Fig. 4C). Poor immunogenicity could prevent the elimination of ELI025 by host antibody responses, and therefore, it could allow ELI025 to act in sterol acquisition inside host tissue.

In conclusion, ELI025 has been successfully cloned and expressed in *E*. *coli*. Genetic, biochemical, and immunological characterization showed that ELI025 is a small glycoprotein, abundantly secreted by *P*. *insidiosum*. ELI025 had two isoforms (glycosylated and non-glycosylated form), and was not recognized by host antibodies. The upstream region of *ELI025* shared core promoter elements with the promoters of other oomycete genes. Among human fungal and oomycete pathogens, ELI025 is unique to *P*. *insidiosum*, and therefore, it is a potential target for development of more specific diagnostic tests. Characterization of ELI025 provided a new insight into the biology and pathogenesis of the understudied microorganism, *P*. *insidiosum*, and it could lead to a discovery of a new strategy for infection control.
